# A Novel Bioactive Glass Containing Therapeutic Ions with Enhanced Biocompatibility

**DOI:** 10.3390/ma13204600

**Published:** 2020-10-15

**Authors:** Rachele Sergi, Devis Bellucci, Roberta Salvatori, Alexandre Anesi, Valeria Cannillo

**Affiliations:** 1 Department of Engineering “Enzo Ferrari”, University of Modena and Reggio Emilia, Via P. Vivarelli 10, 41125 Modena, Italy; rachele.sergi@unimore.it (R.S.); devis.bellucci@unimore.it (D.B.); 2 Biomaterials Laboratory, Department of Medical and Surgical Sciences of Children and Adults, University of Modena and Reggio Emilia, Via Campi 213/A, 41125 Modena, Italy; roberta.salvatori@unimore.it (R.S.); alexandre.anesi@unimore.it (A.A.)

**Keywords:** bioactive glasses, strontium, magnesium, high crystallization temperature, dissolution rate, tissue regeneration, cell culture

## Abstract

A novel bioactive glass containing therapeutic ions with enhanced biocompatibility was designed and produced by the classical melt-quenching route. Starting from a very promising composition (Bio_MS), which combined bioactivity and high crystallization temperature, the ratio between some oxides was tailored to obtain a new and more reactive (in terms of dissolution rate) bioactive glass, called BGMSN (composition in mol%: 6.1 Na_2_O, 31.3 CaO, 5 MgO, 10 SrO, 2.6 P_2_O_5_, 45 SiO_2_). The aim of this work was to produce a bioactive glass with a good biological performance, preserving, at the same time, the high crystallization temperature achieved for Bio_MS; this is strategic in order to avoid undesired crystalline phases during thermal treatments, which can undermine the bioactivity and even the stability of final products. A complete characterization of the novel bioactive glass was performed in terms of thermal, mechanical and biological properties and in vitro bioactivity. The thermal behavior of the bioactive glass was studied by heating microscopy, differential thermal analysis (DTA) and optical dilatometry; BGMSN showed a very high crystallization temperature and a high sinterability parameter, thus being suitable for applications where thermal treatments are required, such as sintered samples, coatings and scaffolds. Mechanical properties were investigated by the micro-indentation technique. The in vitro biological properties were evaluated by means of both direct and indirect cell tests, i.e., neutral red (NR) uptake and MTT assay, using murine long bone osteocyte Y4 (MLO-Y4) cells: the cellular viability of BGMSN was higher compared to cellular viability of 45S5, both in direct and indirect tests. Finally, the in vitro bioactivity test by soaking samples in simulated body fluid (SBF) showed high dissolution rate, with a good rate of formation of hydroxyapatite.

## 1. Introduction

Since the development of the 45S5 Bioglass^®^ (45S5) by L.L. Hench [1,2], bioactive glasses have been widely employed as biomaterials for small bone implants, orthopedic and periodontal reconstruction, scaffolds and coatings. Bioactive glasses should exhibit biocompatibility, non-cytotoxicity, immunogenicity and bioactivity to be suitable for the human body without causing the formation of fibrous tissues in the area or the rejection of implant. The human body requires some elements which are indispensable for the regulation of body fluids and acid-base balance. These elements can be divided in macro-elements and micro-elements. Macro-elements include phosphorous (P), calcium (Ca), chlorine (Cl), sodium (Na) and potassium (K), in the quantity >100 mg/dL; micro-elements are copper (Cu), zinc (Zn), sulphur (S) and magnesium (Mg), <100 mg/dL [3]. Ca and P are the major constituents of bone and teeth. Calcium is utilized by organisms in muscle contraction, membrane permeability and neuromuscular excitability, whereas phosphorous is involved in the synthesis of phosphoproteins and phospholipids. Furthermore, silicon is a component of connective tissue and is involved in the calcification of bones. Sodium is a main cation of extracellular fluid; it regulates plasma volume and preserves osmotic pressure. In this regard, from the compositional aspect of bioactive glasses, certain oxides (i.e., silicon oxide, phosphorous oxide, calcium oxide and sodium oxide) are included in the composition to enhance tissue remodeling to its natural form when bioactive glasses undergo dissolution. These oxides help the bond to bone formation and their dissolution acts on osteoprogenitors cells stimulating new bone growth (i.e., Ca and Si ions have been shown to stimulate osteoblast cell division and production of growth factors (GFs)) [2,4]. The bonding with bone is ascribed to the formation of hydroxycarbonate apatite (HCA) [5]. Such formation is well understood and the mechanism is widely reported in the literature [6,7]. A lot of research work has been carried out to develop bioactive glasses with different compositions to enhance biocompatibility and bioactivity (i.e., the ability to form a HCA layer under biological fluid contact) and to induce a specific response in the host [8]. In recent years, many other applications of bioactive glasses outside the skeletal system have emerged—for example, for tissue engineering (also soft tissues) and wound healing [9,10]. 

Typically, such applications require thermal treatments to obtain products, such as sintered parts, scaffolds and coatings [11,12]. In this scenario, the crystallization behavior is of pivotal importance, since it is well known that crystallization can inhibit, or at least reduce, bioactivity [2] and, in some cases, even undermine the stability of the implant [13]. In fact, 45S5, besides creating a high-pH environment causing cytotoxicity [14], has a high tendency to crystallize, showing a narrow sintering window [2], which represents a limit in case of thermal treatment. Therefore, a high crystallization temperature and low tendency to crystallize are fundamental characteristics to avoid complete or partial crystallization phenomena. 

Recently, a novel bioactive glass called Bio_MS has been developed [15] to achieve a high crystallization temperature: Bio_MS returned very promising results in terms of thermal properties and biological performance, showing cell adhesion, colonization and bone differentiation, being also suitable for possible thermal treatments [15]. 

In this work, a novel bioactive glass named BGMSN was designed, starting from the successful composition of Bio_MS, with the aim to improve its reactivity in terms of biological performance and in vitro bioactivity, preserving, at the same time, high crystallization temperature. The ratio between some oxides was tailored to obtain a new and more reactive (in terms of dissolution rate) bioactive glass, based on the concept of network connectivity [2]. It is well known in the literature that by adding Na_2_O (i.e., a network modifier) at the expense of SiO_2_ (i.e., network forming), the dissolution rate of the bioactive glass increases because its network connectivity decreases [2]. As a matter of fact, a more reactive bioactive glass—in terms of dissolution rate—with a faster release of therapeutic ions could be particularly suitable for regenerative medicine.

The dissolution rate and consequently the rate of formation of HCA depend on the atomic structure of the bioactive glass. It has been shown that the dissolution of SiO_2_ (i.e. network forming) is directly related to the bioactivity of the bioactive glass [16]. In silica-based bioactive glasses, the connectivity of the network depends on the SiO_2_ amount and on other ions that act as network modifiers. By decreasing the SiO_2_ amount, which constitutes the bioactive glass network, and increasing the Na_2_O amount, the dissolution rate of bioactive glass increases because the network connectivity decreases. This increase is due to a lower SiO_2_ amount, which means a less-connected silica network with less bridging oxygen bonds (BO), and a higher Na_2_O amount, which contributes to further decreasing the network connectivity [2], since Na atoms break Si−O bonds to produce non-bridging oxygens (NBOs) [17]. Moreover, the glass transition temperature is reduced as the Na_2_O is increased and this is a reflection of the degree of network disruption [17,18]. A less-connected network results in faster dissolution of bioactive glass, improving reactivity and, in turn, bioactivity [2].

Furthermore, SrO and MgO amounts were kept unchanged from the Bio_MS composition because such oxides have been shown to further contribute to decrease the glass transition temperature T_g_ and increase the crystallization temperature T_c_ [19,20,21,22]. Nonetheless, the incorporation of SrO and MgO not only aims to improve thermal properties but it also aims to enhance the biological performance; it has been reported that Sr and Mg ions improve effectiveness for bone regeneration [23,24,25,26] and cell proliferation [27,28]. 

A complete and thorough characterization in terms of thermal, mechanical and biological properties and in vitro bioactivity of the novel BGMSN was performed to assess the favorable combination of features, i.e., high crystallization temperature and good biological performance. Differential thermal analysis (DTA) and a heating microscope were used to determine the critical temperatures of BGMSN (glass transition temperature, sintering temperature, onset crystallization temperature, peak crystallization temperature and melting temperature). Moreover, optical dilatometry was employed to determine the thermal expansion coefficient (CTE). On the other hand, mechanical properties were assessed by micro-indentation technique to evaluate the Young’s modulus and Vickers hardness of BGMSN.

Furthermore, in vitro bioactivity was studied by soaking samples in simulated body fluid (SBF) solution for 1, 7 and 14 days, in terms of formation of hydroxycarbonate apatite (HCA) on the samples’ surface. Additionally, the biological performance of BGMSN was investigated by direct assay (neutral red (NR) uptake) and indirect assay (MTT) using MLO-Y4 cells.

## 2. Materials and Methods 

### 2.1. Preparation of Glass Powders

The novel composition of BGMSN is reported in Table 1. Commercial raw powders from Carlo Erba Reagenti, Italy, namely SiO_2_, Ca_3_(PO_4_)_2_, SrCO_3_, MgCO_3_, CaCO_3_ and Na_2_CO_3_, were mixed in a lab shaker for 3 h. Then, the mixed powders were melted in a Pt crucible in air to produce BGMSN bioactive glass by a melt-quenching route as described elsewhere [29,30,31,32]. The thermal cycle to melt BGMSN was: (a) 25 °C → 1100 °C, rate 10 °C/min; (b) decarbonation step: 1100 °C for 2 h; (c) 1100 °C →1450 °C, rate 10 °C/min; (d) 1450 °C for 45 min. The molten glass was quickly quenched in water (at room temperature) to obtain a frit. Such frit was dried at 110 °C for 14 h and then ground to obtain powders with final grain size <63 μm. 

### 2.2. Thermal Behaviour of the Bioactive Glass

Heating microscopy (HM, Misura 3.32; Expert System Solutions, Modena, Italy), and differential thermal analysis (DTA, Netzsch Differential Thermal Analyzer STA 429 CD, Netzsch-Gerätebau GmbH, Selb, Germany) techniques were employed to determine the critical temperatures of BGMSN. The heating microscope permits (i) to investigate the critical temperatures for the optimization of manufacturing processes, identifying the sintering temperature (T_s_), softening temperature (T_d_) and melting temperature (T_m_) and (ii) to study the sintering behavior of the glass. On the other hand, during DTA analysis, 30 mg of glass powder was heated from room temperature to 1400 °C (rate 10 °C/min) to obtain the glass transition temperature (T_g_), the onset crystallization temperature (T_c_onset_) and the peak crystallization temperature (T_c_).

Moreover, optical dilatometry (OD) was performed (using Misura 3.32, Expert System Solutions, Modena, Italy) to determine the thermal expansion coefficient (CTE) and to give precious information regarding the sintering kinetics, since sintering is usually accompanied by high specimen shrinkages. A 5 × 5 × 15 mm^3^ bar was heated from 25 °C to 730 °C at 5 °C/min.

The sintering attitude of BGMSN was also evaluated by means of the sinterability parameter (Sc) [33]. Sc expresses the competing mechanisms between crystallization and sintering when the glass powders are heated and it is calculated as follows [33]:

Sc = T_c_onset_ − T_s_

Finally, sintered disks were fabricated by pressing BGMSN powders with some drops of ethanol. Consequently, the disks were sintered at 711 °C (i.e., the determined sintering temperature T_s_) for 3 h. Additionally, 45S5 disks were also prepared following the same procedure to be used as reference during biological tests. 

### 2.3. Microstructural Characterization 

Environmental scanning electron microscopy (ESEM) was used to study the microstructure of the sintered disks (Quanta 2000, FEI Co., Eindhoven, The Netherlands). ESEM was utilized before and after immersion in simulated body fluid (SBF) solution (Section 2.5) to evaluate the formation of hydroxycarbonate apatite (HCA) on the samples’ surface. Furthermore, a disk previously sintered at 711 °C and subsequently crushed in powder was investigated by X-ray diffractometry (XRD, Philips PW3710, Almelo, The Netherlands), to confirm that BGMSN samples remained amorphous despite the thermal treatment. Data collection was performed using a Cu-kα X-ray line by a 2θ scan method in the range of 10–90° with steps of 0.02° (6 s each step).

### 2.4. Mechanical Testing

Mechanical properties were studied by using the micro-indentation technique. Open Platform equipment (CSM Instruments, Peseux, Switzerland) was used to investigate both Young’s modulus and hardness of BGMSN (with Vickers indenter tip). The sintered disks were embedded into epoxy resin and then lapped and polished. A load of 100 mN was used during the indentation process, with a load/unload rate of 200 mN/min. Ten different sintered disks were tested, obtaining fifteen measurements for each sintered disk. The Young’s modulus was calculated from the unloading part of the load–depth curve according to [34]; the load–penetration depth curve was automatically recorded for each indentation.

### 2.5. In Vitro Bioactivity

BGMSN sintered disks were immersed in simulated body fluid (SBF) solution to investigate the in vitro bioactivity. The SBF solution and the volume used during the test were prepared and calculated according to the protocol formulated by Kokubo et al. [35]. The BGMSN sintered disks were soaked in SBF for 1, 7 and 14 days. After each soaking time, the sintered disks were carefully washed with distilled water and then left to dry at room temperature. Then, the hydroxycarbonate apatite (HCA) on the surface of sintered disks was investigated by means of ESEM.

### 2.6. Biological Tests

The biological performance was investigated using MLO-Y4 (murine long bone osteocyte Y4), both in terms of direct and indirect cell tests. 

The in vitro direct assay (NR uptake) and indirect assay (MTT) were performed according to the International Organization for Standardization [36,37,38]. The NR uptake assay is based on the capability of viable cells to bind neutral red (NR) dye. Such capability of cells to bind NR dye decreases in case of alteration at the surface of cells or at the lysosomal membrane. Thus, through the different incorporation of NR dye, viable, damaged or dead cells are distinguished. On the other hand, MTT is an indirect test named after the chemical compound 3- (4,5-Dimethyl-2-thiazolyl) -2,5-diphenyl-2H-tetrazolium bromide, which is reduced to an insoluble product (formazan) only by metabolically active cells; in fact, only in them is it reduced by the mitochondrial succinate dehydrogenase enzyme. Subsequently, a solubilization solution (dimethyl sulfoxide; DMSO) is used to dissolve the formazan product. The formazan concentration into metabolically lively cells is then quantified using a spectrophotometer (Multiscan RC by Thermolab system, Helsinki, Finland).

#### 2.6.1. Culture of MLO-Y4 Cells

Dulbecco’s modified Eagle’s Medium (DMEM)—containing 2 mM L-Glutamine, 1 mM sodium pyruvate, pen-streptomycin and 10% (*v*/*v*) fetal bovine serum (FBS)—was used as medium to grow MLO-Y4 cells. The cells were incubated at 37 ± 1 °C, 5 ± 1% CO_2_/air and 90 ± 5% humidity. Then, cells (5 × 10^3^ cells in 100 µL DMEM each well) were seeded in a multi-well culture plate. Three replicates were prepared and tested for 24 h and 72 h.

#### 2.6.2. Bioactive Glasses’ Eluate Preparation

BGMSN and 45S5 disks were immersed in DMEM with a ratio of 6 cm^2^/mL between the area of disks and the extraction liquid volume. DMEM with non-sterile latex-free glove was used as a positive control (CTRL+) with a ratio of 6 cm^2^/mL between the area of the glove and the extraction liquid volume. On the other hand, as a negative control (CTRL-), DMEM only was used. The centrifuge tubes were incubated at 37 °C for 24 h. Eluates were filtered with 0.22 μm filters (Millex-Gs 0.22 µm, Merck Millipore, Germany) before use.

#### 2.6.3. NR Uptake Assay at 24 h and 72 h 

Cells (MLO-Y4) were cultured in multi-well plates in direct contact with BGMSN sintered disks at 37 ± 1 °C, 5 ± 1% CO_2_/air and 90 ± 5% humidity. After 24 h and 72 h, the morphology of cells was investigated by using an optical microscope. Then, 150 µL of NR solution was added to each well after removing DMEM (i.e., the culture medium). After 3 h of incubation at 37 ± 1 °C, the NR solution was removed and 150 µL pre-warmed Dulbecco’s Phosphate Buffered Saline (DPBS) was used to eliminate the surplus of NR solution. Then, 1500 µL of NR Desorb solution (i.e., the extraction solution) was added to extract NR from cells; the multi-well was incubated for 20 min at room temperature. The NR dye amount incorporated into cells was determined by measuring the absorbance (at 540 nm).

#### 2.6.4. MTT Assay 

MLO-Y4 cells were seeded in multi-wells plates in contact with 50 μL of glasses’ eluate. After 24 h of treatment, 8 µL of tetrazolium salt (5 mg/mL in DPBS) was added to each well and incubated at 37 °C. 3 h after incubation, the formazan was solubilized in 100 μL of dimethyl sulfoxide (DMSO) after removing the media. Then, after fifteen minutes of incubation at 37 °C, the absorbance was measured (at 540 nm) to quantify the amount of newly synthesized formazan.

#### 2.6.5. Statistical Analysis 

Student’s *t*-test was employed to establish the statistical differences among groups (*p* < 0.05), with a two-population comparison.

## 3. Results and Discussion

### 3.1. Thermal Behaviour 

The critical temperatures of BGMSN determined by heating microscopy and DTA are reported in Table 2.

**Figure 1:**
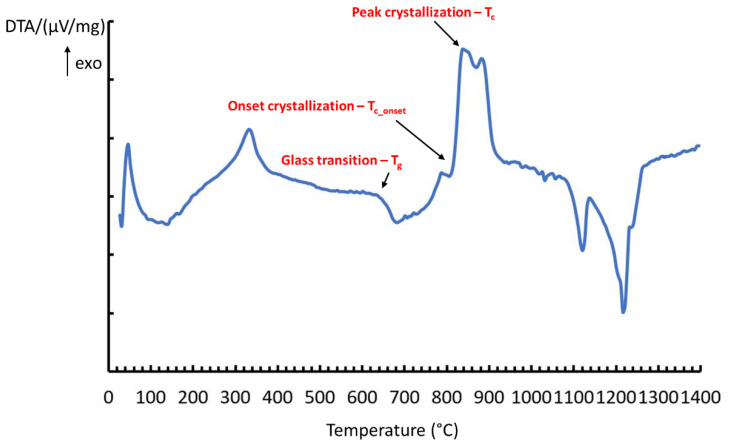
Differential thermal analysis (DTA) curve of BGMSN powders.

The onset of crystallization can be observed at ~806 °C and an exothermic peak, corresponding to the crystallization temperature, was visible between 810 °C and 900 °C (Figure 1). The glass transition temperature (T_g_) of BGMSN was at ~631 °C and its peak crystallization temperature (T_c_) was 851 °C (Figure 1); both temperatures are much higher compared to those of 45S5 [2,39]. 

The glass transition temperature and the crystallization temperature of BGMSN are slightly lower compared to those of Bio_MS [15] (Table 2) because the higher amount of Na_2_O oxide (i.e., a network modifier) in BGMSN resulted in the decrease in the link of non-bridging oxygen bond [17,18]. Despite this, BGMSN has one of the highest crystallization temperatures ever reported for a bioactive glass.

Nevertheless, BGMSN has much more favorable characteristic temperatures compared to commonly used bioactive glasses, such as 45S5. To this regard, it has been discovered that SrO and MgO decrease the T_g_ and increase T_c_ of bioactive glasses [19,20,21]. The decrease in T_g_ of SrO-containing bioactive glasses was ascribed to the ionic radius of the Sr ion, which is larger than that of the Ca ion. The larger ionic radius of Sr ion causes an expansion of the glass network and non-bridging oxygens (NBO) are less attracted [19]. Additionally, MgO also contributes to the decreased T_g_ of silicate-based bioactive glasses, as reported in [20,21]. The effect of MgO addition could be due to the lower average bond strength of Si-O-Mg compared to that shown by Si-O-Si bonds. Furthermore, the specific role of MgO in silicate-based bioactive glasses’ network is still unclear; indeed, it has been reported to act both as a network modifier [40] and as an intermediate modifier [20,41]. MgO exerts an inhibitory effect on crystallization with a consequent increase in T_c_, as reported in the literature [42,43]. 

A good thermal behavior of bioactive glasses can be also determined by the glass stability parameter (GS), which is the resistance against devitrification after reheating. GS is characterized by the difference between the peak crystallization temperature and the glass transition temperature [44]. GS can be also evaluated by using the parameter proposed by Hrubý [45] or by the T_g_/T_m_ ratio; i.e., the glass transition temperature divided by the melting temperature. The larger this ratio is, the higher the GS is [46,47]. The T_g_/T_m_ ratio is 0.530 for BGMSN and 0.529 for Bio_MS. Therefore, GS seems slightly more advantageous for BGMSN than for Bio_MS. 

Moreover, the sinterability parameter (Sc), which can be considered as the competition of the sintering ability versus the crystallization tendency of glass powders [33], was higher for BGMSN (compared to Bio_MS [15]), as reported in Table 2.

Therefore, even if the crystallization temperature is slightly higher for Bio_MS than for BGMSN, indeed, BGMSN seems to have more favorable characteristics in terms of thermal properties. Thus, BGMSN seems perfectly suitable for applications that require a thermal treatment. 

**Figure 2:**
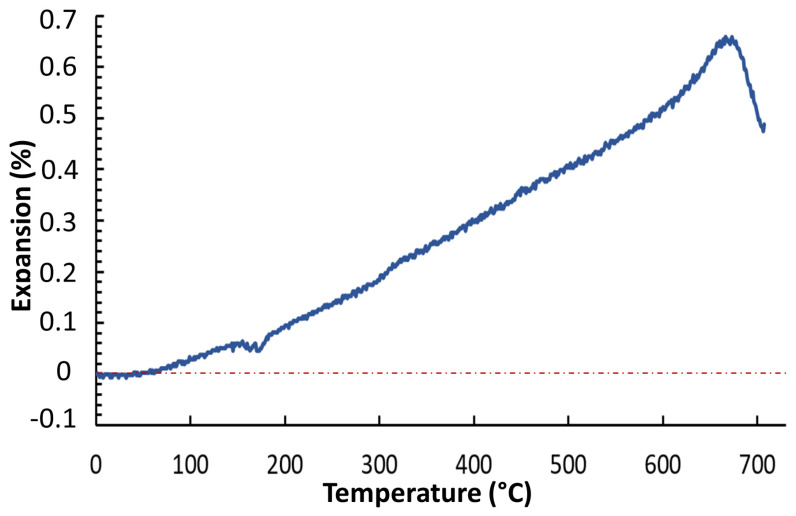
Thermo–dilatometric curve of BGMSN.

Furthermore, Figure 2 shows the optical dilatometer analysis up to 730 °C. From this curve, the CTE was evaluated for different temperature ranges and it resulted as follows: (i) range 50–300 °C, α = 6.50 × 10^−6^ °C^−1^; (ii) range 300–600 °C, α = 10.85 × 10^−6^ °C^−1^; (iii) range 600–700 °C, α = 15.74 × 10^−6^ °C^−1^. The average value in the range 50–600°C was 8.87 × 10^−6^ °C^−1^. The CTE of BGMSN was low compared to that of 45S5, which is 15 × 10^−6^ °C^−1^ (in the range 200–400 °C), as reported elsewhere [2,48]. 

The CTE is very important for bioactive glasses, especially in the case that they have to be applied as coatings on metallic implants [49]. In fact, due to the CTE mismatch between the coating and the substrate, thermal residual stresses may arise [48,50,51,52] and this can even compromise the stability of the coating and its adhesion to the implant [52]. 

It is worth noting that the CTE value of BGMSN is comparable to the CTE of Ti6Al4V (9.5–10.5 × 10^−6^ °C^−1^ at 400 °C); thus, BGMSN could be suitable for coatings at temperatures below the α → β transformation of Ti [48].

### 3.2. Microstructural Characterization 

**Figure 3:**
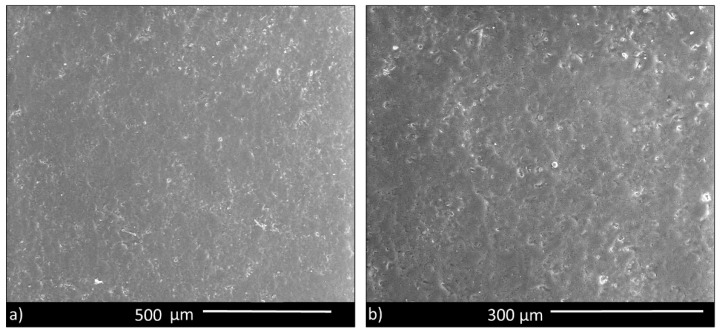
(**a**) BGMSN sintered disk, showing a good densification; (**b**) higher magnification image.

SEM images of the surface of sintered disks (Figure 3) showed a dense microstructure, apart from some small residual pores. Therefore, BGMSN appeared well sintered, confirming that the sintering temperature (711 °C) was adequate to consolidate the samples. 

**Figure 4:**
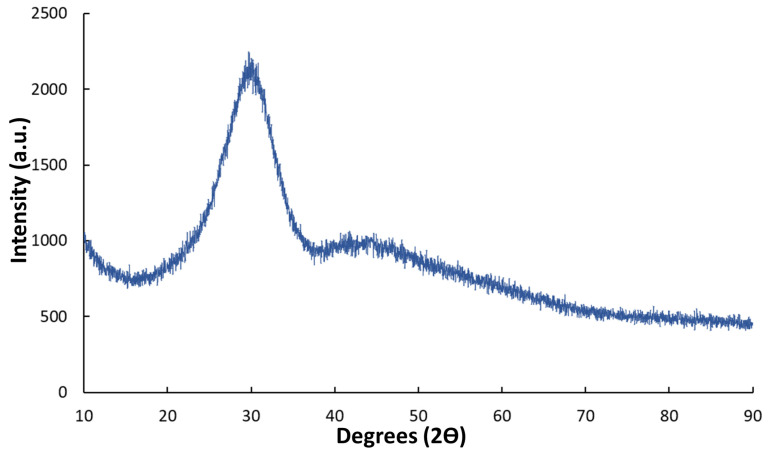
XRD diffractogram of BGMSN sintered samples.

Additionally, XRD analysis confirmed the amorphous nature of BGMSN disks after the sintering process. Indeed, the XRD diffractogram acquired on sintered disks (crushed into powder) was characterized by the typical shape of amorphous glasses (Figure 4). The absence of any crystallization peak in the diffractogram confirmed that the sintering temperature (711 °C) was appropriate and that BGMSN has a good glass stability (GS), as already discussed above (Section 3.1).

### 3.3. Mechanical Characterization

The mechanical properties of BGMSN sintered disks were investigated by the micro-indentation technique. The Vickers hardness of sintered BGMSN was 727.4 ± 163 HV (Table 3).

The value of hardness is higher compared to the value of sintered BG_Ca-Mix bioactive glass (564 ± 47 HV) [53]. The increase in the hardness could be due to the presence of MgO and SrO in the composition of BGMSN, because of their high strength of metal-oxygen bond [54]. Furthermore, the elastic modulus of BGMSN was 94 ± 13 GPa, which was higher compared to that of 45S5 (35 GPa) [2,55,56]. This confirms that the addition of MgO and SrO in its composition favors thermal properties as well as mechanical properties. Furthermore, these results corroborate that BGMSN disks were well sintered, as previously observed in Figure 3, and thus, the sintering temperature reported in Table 2 is suitable for the realization of compact samples.

### 3.4. In Vitro Bioactivity Investigation 

**Figure 5:**
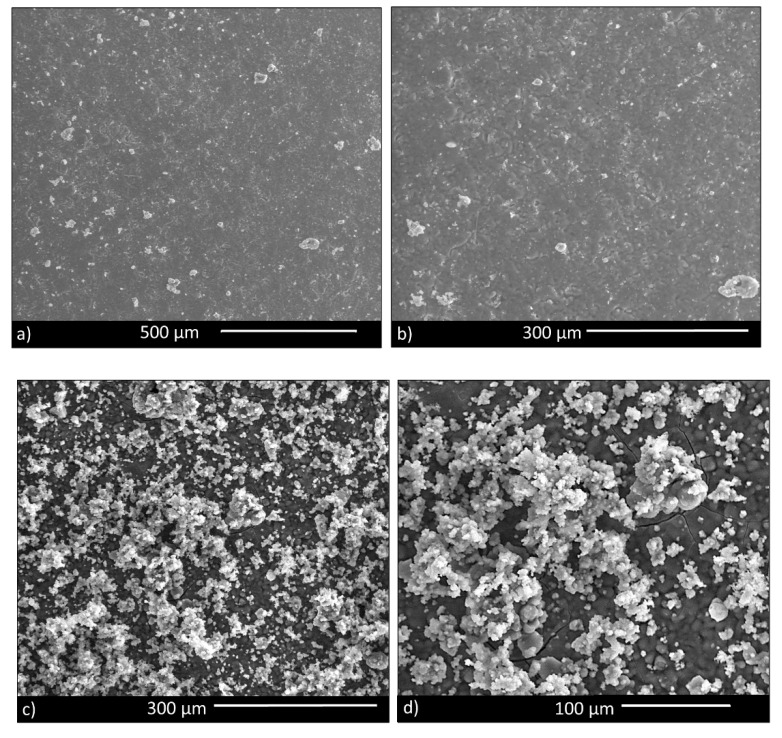

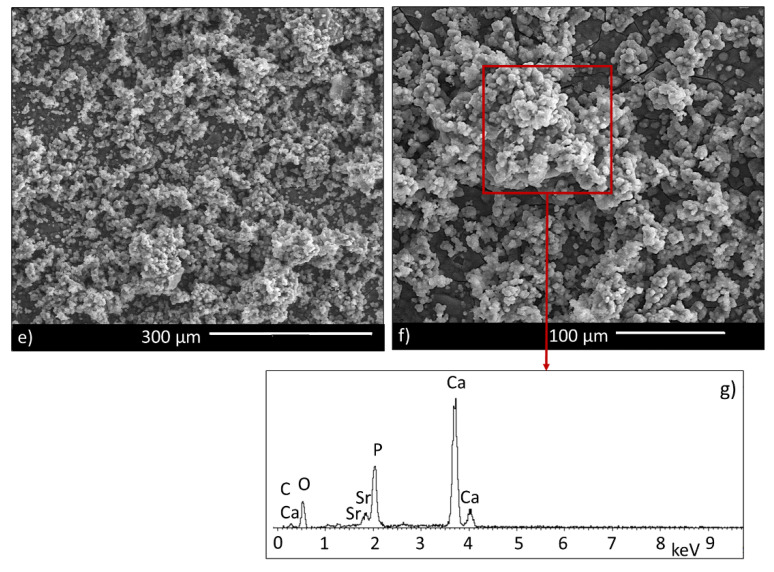
Hydroxycarbonate apatite formation on samples’ surfaces after 1 day (**a**,**b**), 7 days (**c**,**d**) and 14 days (**e**,**f**) and EDS spectrum (**g**) after immersion in simulated body fluid (SBF) solution.

ESEM analyses were performed on BGMSN disks 1, 7 and 14 days after immersion in SBF. One day after immersion in SBF, some globular precipitates of hydroxycarbonate apatite (HCA) were visible on the disks’ surfaces (Figure 5a,b), while 7 days after immersion, the disks’ surfaces were completely covered by precipitates of HCA with their typical morphology (i.e., cauliflower) (Figure 5c,d). Furthermore, 14 days after immersion, the surface of the BGMSN disks was fully covered by precipitates of HCA (Figure 5e,f). The EDS analysis reported in Figure 5g confirms the presence of HCA on the surface of the BGMSN disks. Hydroxycarbonate apatite (HCA) mimics the mineral component of bone and bioactive glasses bond to bone via the formation of a superficial HCA layer. Therefore, the ability of hydroxyapatite to nucleate on the disks’ surfaces could be a preliminary estimation of the bone-bonding ability of BGMSN. These findings ascertain the high reactivity of BGMSN in terms of in vitro bioactivity. 

### 3.5. Biological Investigation

**Figure 6:**
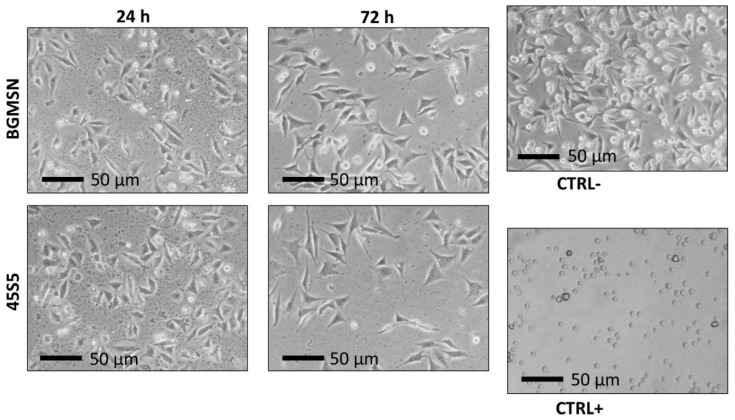
Morphological evaluation of murine long bone osteocyte Y4 (MLO-Y4) cells 24 h and 72 h after seeding in direct contact with sintered disks using optical microscopy (Nikon TMF, Tokyo, Japan) 50× magnification.

Biological tests were performed both on BGMSN and 45S5 (reference material) sintered disks. After seeding in direct contact for 24 and 72 h, MLO-Y4 cells did not show significant changes in cellular morphology, such as lysing, rounding, etc. (Figure 6). The MLO-Y4 cells grew on BGMSN sintered disks, developing a morphology similar to the corresponding cells grown on 45S5 sintered disks and CTRL- (Figure 6).

**Figure 7:**
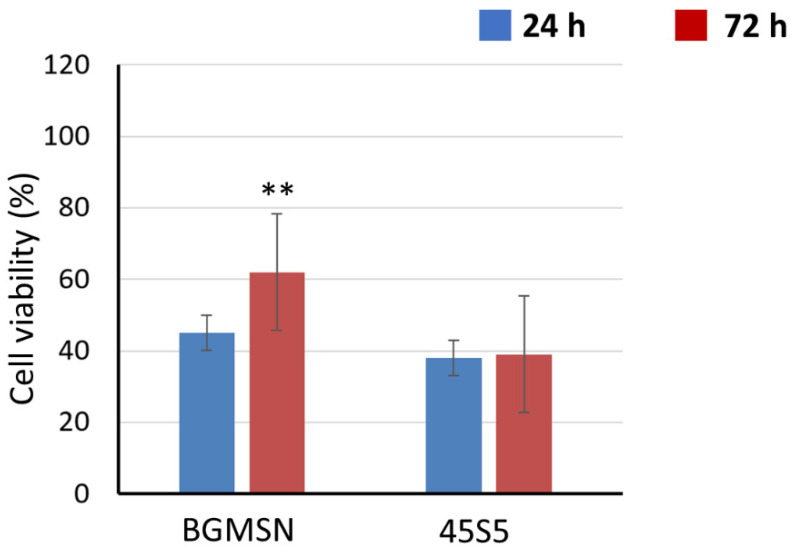
Neutral red (NR) uptake 24 h and 72 h after seeding using MLO-Y4. ** *p* < 0.05.

At 24 h after direct contact, the cellular viability value of BGMSN was higher compared to the cellular viability of 45S5, as shown in Figure 7. The cellular viability assesses the non-cytotoxicity of BGMSN and no significant decrease in lysosomal activity imputable to cytotoxicity was observed [57]. NR uptake was also performed at 72 h after seeding; the cellular viability of BGMSN was significantly higher (*p* < 0.05) compared to the cellular viability of 45S5, as reported in Figure 7.

This could be due to the addition of SrO and MgO in the BGMSN composition with respect to 45S5; such elements are known to have beneficial biological properties, as mentioned earlier. In fact, Sr and Mg ions are widely known to improve the replication of pre-osteoblastic cells [24,26,58] as well as cell proliferation and angiogenesis [59,60]. 

Additionally, the dissolution also permits the releasing of ions such as Si, Ca and P, which are known to positively influence cell viability and bone regeneration [61,62]. Si ions influence the metabolic process of bone forming. Furthermore, Si is known to reduce bone resorption due to calcium deficiency, to increase bone density [63] and to induce collagen I production and osteoblast differentiation in human osteoblast cells [61]. On the other hand, Ca showed various roles in cells and living systems [64], being particularly relevant in bone tissue [65] since it is one of the more essential components of the mineralized bone matrix, together with phosphate [66]. Furthermore, Ca ions induce the proliferation of mesenchymal precursors cells and of mature bone cells as well [67]. Finally, P ions are not only involved in various physiological process (i.e., cellular signaling, acid-base homeostasis and in maintaining the integrity of cell membrane) but they also regulate the differentiation and mineralization of osteoblasts and pre-osteoblasts [68,69]. These ions in vivo are fundamental in stimulating bone regeneration, as reported in the literature, and, in fact, in vitro tests showed good cellular viability (Figure 7) due to their synergic effect. 

**Figure 8:**
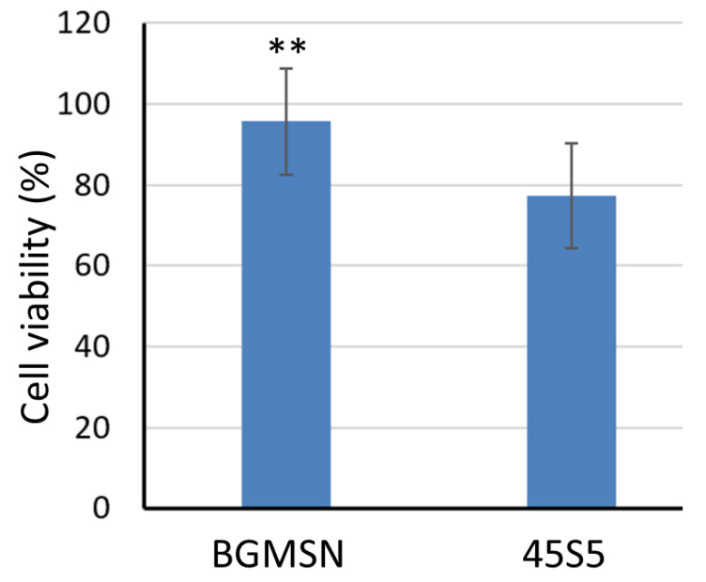
MTT test 24 h after culturing MLO-Y4 cells in contact with samples’ eluates. ** *p* < 0.05.

Furthermore, MTT assay was performed (24 h after seeding), as shown in Figure 8. The cellular viability of BGMSN was significantly higher (*p* < 0.05) compared to that of 45S5. Therefore, the positive effect pointed out by the NR test was confirmed also by the indirect test. 

In conclusion, the good cell morphology, observed in Figure 6, along with the results from NR test and MTT assay confirm the non-cytotoxicity of BGMSN and its good biological performance, with better cell viability compared to 45S5. It can be hypothesized that the enhanced biological performance of BGMSN with respect to 45S5 depends on the presence of Sr and Mg in its composition, which improves cell proliferation, angiogenesis and replication of pre-osteoblastic cells. On the other hand, it should be noted that the 45S5 glass undergoes crystallization during the thermal treatment to sinter the disks; on the contrary, BGMSN remained completely amorphous. Therefore, it seems reasonable to think that the reduced biological performance observed for 45S5 samples can be ascribed, also, to the glass crystallization.

Nevertheless, further studies to deeper investigate the biological potential of BGMSN are in progress. In vivo animal experiments are already underway, to compare the biological potential of BGMSN to 45S5 and Bio_MS; the in vivo in animal model would study glass behavior (i.e., bioactive glass dissolution in long-term implants in contact with body fluids) and the bone regeneration process. Furthermore, an innovative 3D cellular model, able to mimic the potential clinical application of a given materials and used for Bio_MS [15], could be utilized to further test BGMSN.

## 4. Conclusions

In this work, a new bioactive glass was designed, produced and characterized by modifying the composition from a previous bioactive glass (Bio_MS), which showed a very good combination of properties, namely biological performance and high crystallization temperature. The composition of BGMSN was tailored to improve bioactive glass reactivity, bioactivity in terms of rate of HCA formation and biological properties, maintaining, at the same time, a very high crystallization temperature. 

With regard to thermal behavior, BGMSN showed excellent properties, displaying a very high crystallization temperature and a sinterability parameter (Sc) even higher than Bio_MS. Thus, BGMSN can be considered among the most promising bioactive glasses in terms of thermal behavior. 

The high values of hardness and Young’s modulus obtained by the micro-indentation technique confirmed that BGMSN has adequate mechanical properties. 

Furthermore, Sr and Mg ions not only favor BGMSN sinterability, high crystallization temperature and mechanical properties, but such ions may also have beneficial effects in terms of biological properties, as widely reported in the literature. The biological performance of BGMSN was assessed using MLO-Y4 cells. As a matter of fact, BGMSN showed a higher cellular viability compared to that of 45S5, which is considered as the gold standard.

In conclusion, BGMSN showed good biological properties together with a high crystallization temperature and a very good sinterability parameter, making it promising for orthopedic and dentistry applications, which usually require thermal treatments. In fact, BGMSN could be favorably employed to fabricate devices, such as sintered bodies, scaffolds and coatings.

In vivo animal models will represent future investigations to confirm the positive and encouraging results obtained in this work. 

## Figures and Tables

**Table 1:** Composition (in oxides mol %) of produced bioactive glass.

Oxides	Na_2_O	CaO	MgO	SrO	P_2_O_5_	SiO_2_
Composition (mol%)	6.1	31.3	5	10	2.6	45

**Table 2:** Characteristic temperatures of BGMSN. * Sc = T_c-onset_ − T_s_.

Sample Name	T_g_ (°C)	T_c-onset_ (°C)	T_c_ (°C)	T_s_ (°C)	T_m_ (°C)	Sc (°C) *
BGMSN	631	806	851	711	1189	95

**Table 3:** Young’s modulus and hardness, as determined by micro-indentation tests.

Young’s Modulus (GPa)	Hardness (Vickers)
94 ± 13	727.4 ± 163

## References

[1] Greenspan D.C. (2016). Glass and Medicine: The Larry Hench Story. Int. J. Appl. Glass Sci..

[2] Jones J.R. (2015). Reprint of: Review of bioactive glass: From Hench to hybrids. Acta Biomater..

[3] Kaur G., Pandey O., Singh K., Homa D., Scott B., Pickrell G. (2013). A review of bioactive glasses: Their structure, properties, fabrication and apatite formation. J. Biomed. Mater. Res. Part A.

[4] Hench L.L. (2002). Third-Generation Biomedical Materials. Science.

[5] Hench L.L., Paschall H.A. (1973). Direct chemical bond of bioactive glass-ceramic materials to bone and muscle. J. Biomed. Mater. Res..

[6] Maçon A.L.B., Kim T.B., Valliant E.M., Goetschius K., Brow R.K., Day D.E., Hoppe A., Boccaccini A.R., Kim I.Y., Ohtsuki C. (2015). A unified in vitro evaluation for apatite-forming ability of bioactive glasses and their variants. J. Mater. Sci. Mater. Med..

[7] Peitl O., Dutra E., Hench L. (2001). L. Highly bioactive P_2_O_5_-Na_2_O-CaO-SiO_2_ glass-ceramics. J. Non-Cryst. Solids.

[8] Baino F., Hamzehlou S., Kargozar S. (2018). Bioactive Glasses: Where Are We and Where Are We Going?. J. Funct. Biomater..

[9] Baino F., Novajra G., Miguez-Pacheco V., Boccaccini A.R., Vitale-Brovarone C. (2016). Bioactive glasses: Special applications outside the skeletal system. J. Non-Cryst. Solids.

[10] Miguez-Pacheco V., Hench L.L., Boccaccini A.R. (2015). Bioactive glasses beyond bone and teeth: Emerging applications in contact with soft tissues. Acta Biomater..

[11] Henao J., Poblano-Salas C., Monsalve M., Corona-Castuera J., Barceinas-Sanchez O. (2019). Bio-active glass coatings manufactured by thermal spray: A status report. J. Mater. Res. Technol..

[12] Baino F., Verné E. (2017). Glass-based coatings on biomedical implants: A state-of-the-art review. Biomed. Glass.

[13] Filho O.P., Latorre G.P., Hench L.L. (1996). Effect of crystallization on apatite-layer formation of bioactive glass 45S5. J. Biomed. Mater. Res..

[14] Fernandes H.R., Gaddam A., Rebelo A., Brazete D., Stan G.E., Ferreira J.M.F. (2018). Bioactive Glasses and Glass-Ceramics for Healthcare Applications in Bone Regeneration and Tissue Engineering. Materials.

[15] Bellucci D., Veronesi E., Dominici M., Cannillo V. (2020). A new bioactive glass with extremely high crystallization temperature and outstanding biological performance. Mater. Sci. Eng. C.

[16] Arcos D., Greenspan D.C., Vallet-Regí M. (2003). A new quantitative method to evaluate thein vitrobioactivity of melt and sol-gel-derived silicate glasses. J. Biomed. Mater. Res. Part A.

[17] Jabraoui H., Vaills Y., Hasnaoui A., Badawi M., Ouaskit S. (2016). Effect of Sodium Oxide Modifier on Structural and Elastic Properties of Silicate Glass. J. Phys. Chem. B.

[18] Cai Y., Su Y., Zhao L., Cui Z. Algorithm Design of Push Service for Telemedicine System. Proceedings of the 2018 International Conference on Robots & Intelligent System (ICRIS).

[19] Lotfibakhshaiesh N., Brauer D.S., Hill R.G. (2010). Bioactive glass engineered coatings for Ti6Al4V alloys: Influence of strontium substitution for calcium on sintering behaviour. J. Non-Cryst. Solids.

[20] Watts S., Hill R., O’Donnell M., Law R. (2010). Influence of magnesia on the structure and properties of bioactive glasses. J. Non-Cryst. Solids.

[21] Vernè E., Bretcanu O., Balagna C., Bianchi C.L., Cannas M., Gatti S., Vitale-Brovarone C. (2008). Early stage reactivity and in vitro behavior of silica-based bioactive glasses and glass-ceramics. J. Mater. Sci. Mater. Med..

[22] Bellucci D., Sola A., Salvatori R., Anesi A., Chiarini L., Cannillo V. (2017). Role of magnesium oxide and strontium oxide as modifiers in silicate-based bioactive glasses: Effects on thermal behaviour, mechanical properties and in-vitro bioactivity. Mater. Sci. Eng. C.

[23] Yang F., Yang D., Tu J., Zheng Q., Cai L., Wang L. (2011). Strontium Enhances Osteogenic Differentiation of Mesenchymal Stem Cells and In Vivo Bone Formation by Activating Wnt/Catenin Signaling. Stem Cells.

[24] Bonnelye E., Chabadel A., Saltel F., Jurdic P. (2008). Dual effect of strontium ranelate: Stimulation of osteoblast differentiation and inhibition of osteoclast formation and resorption in vitro. Bone.

[25] Li Y., Matinmanesh A., Curran D.J., Schemitsch E.H., Zalzal P., Papini M., Wren A.W., Towler M.R., Alhalawani A.M. (2017). Characterization and fracture property of different strontium-containing borate-based glass coatings for Ti6Al4V substrates. J. Non-Cryst. Solids.

[26] Aydın H. (2012). Magnesium Supplementation and Bone. Magnes. Hum. Health Dis..

[27] Bellucci D., Cannillo V. (2018). A novel bioactive glass containing strontium and magnesium with ultra-high crystallization temperature. Mater. Lett..

[28] Li Y., Placek L.M., Coughlan A., Laffir F.R., Pradhan D., Mellott N.P., Wren A.W. (2015). Investigating the influence of Na^+^ and Sr^2+^ on the structure and solubility of SiO_2_–TiO_2_–CaO–Na_2_O/SrO bioactive glass. J. Mater. Sci. Mater. Electron..

[29] Bellucci D., Cannillo V., Sola A. (2011). A new potassium-based bioactive glass: Sintering behaviour and possible applications for bioceramic scaffolds. Ceram. Int..

[30] Bellucci D., Cannillo V., Sola A. (2011). Calcium and potassium addition to facilitate the sintering of bioactive glasses. Mater. Lett..

[31] Bellucci D., Cannillo V., Ciardelli G., Gentile P., Sola A. (2010). Potassium based bioactive glass for bone tissue engineering. Ceram. Int..

[32] Bellucci D., Cannillo V., Sola A. (2011). A New Highly Bioactive Composite for Scaffold Applications: A Feasibility Study. Materials.

[33] Lara C., Pascual M., Durán A. (2004). Glass-forming ability, sinterability and thermal properties in the systems RO–BaO–SiO_2_ (R = Mg, Zn). J. Non-Cryst. Solids.

[34] Oliver W., Pharr G. (1992). An improved technique for determining hardness and elastic modulus using load and displacement sensing indentation experiments. J. Mater. Res..

[35] Kokubo T., Takadama H. (2006). How useful is SBF in predicting in vivo bone bioactivity?. Biomaterials.

[36] ISO 10993-1 (2009). International Organization for Standardization. Biological Evaluation of Medical Devices—Part 1: Evaluation and Testing within a Risk Management Process.

[37] ISO 10993-5 (2009). International Organization for Standardization. Biological Evaluation of Medical Devices—Part 5: Tests for in vitro Cytotoxicity.

[38] ISO 10993-12 (2012). International Organization for Standardization. Biological Evaluation of Medical Devices—Part 12: Sample Preparation and Reference Materials.

[39] Bretcanu O., Chatzistavrou X., Paraskevopoulos K., Conradt R., Thompson I., Boccaccini A.R. (2009). Sintering and crystallisation of 45S5 Bioglass^®^ powder. J. Eur. Ceram. Soc..

[40] George A.M., Stebbins J.F. (1998). Structure and dynamics of magnesium in silicate melts; a high-temperature 25 Mg NMR study. Am. Miner..

[41] Shimoda K., Tobu Y., Hatakeyama M., Nemoto T., Saito K. (2007). Structural investigation of Mg local environments in silicate glasses by ultra-high field 25Mg 3QMAS NMR spectroscopy. Am. Miner..

[42] Ma J., Chen C., Wang D., Jiao Y., Shi J. (2010). Effect of magnesia on the degradability and bioactivity of sol–gel derived SiO_2_–CaO–MgO–P_2_O_5_ system glasses. Colloids Surf. B Biointerfaces.

[43] Ma J., Chen C., Wang D., Meng X.G., Shi J.Z. (2010). In vitro degradability and bioactivity of mesoporous CaO-MgO-P_2_O_5_-SiO_2_ glasses synthesized by sol–gel method. J. Sol-Gel. Sci. Technol..

[44] Shelby J.E. (2007). Introduction to Glass Science and Technology.

[45] Hrubý A. (1972). Evaluation of glass-forming tendency by means of DTA. Czechoslov. J. Phys..

[46] Zheng Q., Zhang Y., Montazerian M., Gulbiten O., Mauro J.C., Zanotto E.D., Yue Y. (2019). Understanding Glass through Differential Scanning Calorimetry. Chem. Rev..

[47] O’Donnell M.D., Candarlioglu P.L., Miller C.A., Gentleman E., Stevens M. (2010). Materials characterisation and cytotoxic assessment of strontium-substituted bioactive glasses for bone regeneration. J. Mater. Chem..

[48] Lopez-Esteban S., Saiz E., Fujino S., Oku T., Suganuma K., Tomsia A. (2003). Bioactive glass coatings for orthopedic metallic implants. J. Eur. Ceram. Soc..

[49] Sergi R., Bellucci D., Cannillo V. (2020). A Comprehensive Review of Bioactive Glass Coatings: State of the Art, Challenges and Future Perspectives. Coatings.

[50] Vitale-Brovarone C., Vernè E. (2005). SiO_2_-CaO-K_2_O coatings on alumina and Ti6Al4V substrates for biomedical applications. J. Mater. Sci. Mater. Electron..

[51] Fujino S., Tokunaga H., Hata S., Saiz E., Tomsia A.P. (2005). Graded glass coatings for Co-Cr implant alloys. J. Mater. Sci..

[52] Cannillo V., Montorsi M., Siligardi C., Sola A., De Portu G., Micele L., Pezzotti G. (2006). Microscale computational simulation and experimental measurement of thermal residual stresses in glass–alumina functionally graded materials. J. Eur. Ceram. Soc..

[53] Bellucci D., Sola A., Cannillo V. (2012). Low Temperature Sintering of Innovative Bioactive Glasses. J. Am. Ceram. Soc..

[54] Kaur G., Pickrell G., Kumar V., Pandey O.P., Singh K., Arya S.K. (2015). Mechanical, dielectric and optical assessment of glass composites prepared using milling technique. Bull. Mater. Sci..

[55] Gerhardt L.-C., Boccaccini A. (2010). Bioactive Glass and Glass-Ceramic Scaffolds for Bone Tissue Engineering. Materials.

[56] Thompson I.D., Hench L.L. (1998). Mechanical properties of bioactive glasses, glass-ceramics and composites. Proc. Inst. Mech. Eng. Part H J. Eng. Med..

[57] Borenfreund E., Puerner J.A. (1985). A simple quantitative procedure using monolayer cultures for cytotoxicity assays (HTD/NR-90). J. Tissue Cult. Methods.

[58] Lijuan X., Liuyun J., Lixin J., Chengdong X. (2013). Synthesis of Mg-substituted hydroxyapatite nanopowders: Effect of two different magnesium sources. Mater. Lett..

[59] Gorustovich A.A., Roether J.A., Boccaccini A.R. (2010). Effect of Bioactive Glasses on Angiogenesis: A Review of In Vitro and In Vivo Evidences. Tissue Eng. Part B Rev..

[60] Yu H., Peng J., Xu Y., Chang J., Li H. (2015). Bioglass Activated Skin Tissue Engineering Constructs for Wound Healing. ACS Appl. Mater. Interfaces.

[61] O’Neill E., Awale G., Daneshmandi L., Umerah O., Lo K.W.-H. (2018). The roles of ions on bone regeneration. Drug Discov. Today.

[62] Rabiee S.M., Nazparvar N., Azizian M., Vashaee D., Tayebi L. (2015). Effect of ion substitution on properties of bioactive glasses: A review. Ceram. Int..

[63] Hoppe A., Güldal N.S., Boccaccini A.R. (2011). A review of the biological response to ionic dissolution products from bioactive glasses and glass-ceramics. Biomaterials.

[64] Clapham D.E. (2007). Calcium Signaling. Cell.

[65] Maeno S., Niki Y., Matsumoto H., Morioka H., Yatabe T., Funayama A., Toyama Y., Taguchi T., Tanaka J. (2005). The effect of calcium ion concentration on osteoblast viability, proliferation and differentiation in monolayer and 3D culture. Biomaterials.

[66] Peacock M. (2010). Calcium Metabolism in Health and Disease. Clin. J. Am. Soc. Nephrol..

[67] Riddle R.C., Taylor A.F., Genetos D.C., Donahue H.J. (2006). MAP kinase and calcium signaling mediate fluid flow-induced human mesenchymal stem cell proliferation. Am. J. Physiol. Physiol..

[68] Penido M.G.M.G., Alon U.S. (2012). Phosphate homeostasis and its role in bone health. Pediatr. Nephrol..

[69] Julien M., Khoshniat S., Lacreusette A., Gatius M., Bozec A., Wagner E.F., Wittrant Y., Masson M., Weiss P., Beck L. (2009). Phosphate-Dependent Regulation of MGP in Osteoblasts: Role of ERK1/2 and Fra-1. J. Bone Miner. Res..

